# Untargeted Metabolomics and Transcriptomics Reveal the Mechanism of Metabolite Differences in Spring Tender Shoots of Tea Plants of Different Ages

**DOI:** 10.3390/foods11152303

**Published:** 2022-08-02

**Authors:** Cuinan Yue, Hua Peng, Wenjin Li, Zhongfei Tong, Zhihui Wang, Puxiang Yang

**Affiliations:** 1 Jiangxi Cash Crops Research Institute, Nanchang 330202, China; m18306083532@163.com (C.Y.); ghost20066@126.com (H.P.); jxlwjin@163.com (W.L.); tzf85023009@126.com (Z.T.); wzh1246900265@163.com (Z.W.); 2 Jiangxi Key Laboratory of Tea Quality and Safety Control, Nanchang 330202, China; 3 Jiangxi Sericulture and Tea Research Institute, Nanchang 330202, China

**Keywords:** tea plant, cultivar, plant age, metabolomics, transcriptomics

## Abstract

The metabolites in the tender shoots of the tea plant are the material basis for the determination of tea quality. The composition and abundance of these metabolites are affected by many key factors, and the tea plant’s age is one of them. However, the effect of plant age on the tender shoot metabolites of tea cultivars of different genotypes is poorly understood. Therefore, we used a combination of untargeted metabolomics and transcriptomics to analyze the differential mechanism behind the differences in the metabolites of the spring tender shoots of 7- and 40-year-old tea plants of two tea cultivars of different genotypes. We found that plant age could significantly change the metabolites in the spring tender shoots of tea plants and that flavonoids, and amino acids and their derivatives, were predominant among the differential metabolites. The quantities of most flavonoids in the aged tea plants of different genotypes were upregulated, which was caused by the upregulated expression of differential genes in the flavonoid biosynthesis pathway. We further discovered that 11 key structural genes play key regulatory roles in the changes in the flavonoid contents of tea plants of different plant ages. However, the influence of plant age on amino acids and their derivatives might be cultivar-specific. By characterizing and evaluating the quality-related metabolites of tea cultivars of two different genotypes at different plant ages, we found that whether an old tea plant (40 years old) can produce high-quality tea is related to the genotype of the tea plant.

## 1. Introduction

The tea plant (*Camellia sinensis* (L.) *O. Kuntze*) belongs to the Theaceae family *Camellia* genus, and is a perennial plant used as a cash crop [1,2]. It originates in the southwest of China [3]. Due to the introduction of tea plants by people from different countries over thousands of years, it has been planted in more than 60 countries and regions across 5 continents [4]. Tea plants, once planted, require many years of management, and allow for many years of harvesting and numerous benefits [5]. The production life of properly managed and cultivated tea gardens can exceed 100 years [6]. However, it is worth noting that the age of the tea plant has a great impact on the maximum yield of tea, which is the best between 20 and 40 years and then decreases [7]. The yield and quality of tea is the main carrier of its economic benefits [5]. Therefore, the influence of the tea plant’s age on tea quality has become an important topic that should be paid attention to.

Tea is made from the tender shoots of the tea plant [8]. Using different processing techniques, the same fresh leaves can be processed into six different types of tea, including green tea, black tea, Oolong tea, white tea, dark tea, and yellow tea [9,10]. As an important raw material for tea processing, the content and composition of the metabolites in the tender shoots are the decisive factors of tea quality [11]. The quality-related metabolites in the tender shoots of tea plants mainly include catechins, flavonoids and their glycosides, amino acids, alkaloids, nucleotides, saccharides, and organic acids [12,13]. Catechins, flavonoids and their glycosides, and alkaloids are mainly bitter and astringent, while amino acids, nucleotides, and saccharides are mainly umami and sweet [14]. If the content of metabolites with umami and sweet tastes in the tender shoots is high and the content of metabolites with bitter and astringent tastes is low, higher-quality tea products can be made with these raw materials [8,15]. There are many factors that affect the content and composition of quality-related metabolites in tender shoots, and the tea plant’s age is one of these key factors [16]. With the continuous aging of the tea plant, the accumulation of nutritional elements and quality components changes, resulting in differences in the quality of the processed tea [17]. Previous studies have shown that there are obvious metabolic differences between the tender shoots of tea plants at different ages, and some metabolites are uniquely dependent on the age of the tea plants [18,19]. In addition, the plant’s age changes the metabolism of the phenols and amino acids in the tea leaves, which is regulated by key genes in their biosynthesis pathways [20]. Although some effects of plant age on tea quality have been reported, in contrast to the depth of research regarding relevant factors, such as environment and processing [13,21,22], research on the influence mechanism of plant age on the metabolites of the tender shoots of tea is seriously lagging. The main reason is that the sample materials from the same tea cultivar but with plants of different ages planted in the same environment and under the same management mode are very difficult to find, which has restricted the exploration of its mechanism.

Omics technology provides a powerful tool for tea plant research, which is characterized by its high throughput, high sensitivity, and systematic nature [23]. Metabolomics can be used for qualitative and quantitative analysis of low-molecular-weight substances produced in tea plants in a specific period [24]. Transcriptomics can study the regularities of gene expression, transcription, and regulation in tea plant cells in a specific period from the whole RNA level [25]. The combination of these two technologies has successfully solved many difficult problems in tea research, such as the regulation network of flavonoids in different seasons [26], the accumulation mechanism of anthocyanins in tea plants with purple bud and leaf [27], the key genes of catechin metabolism [28], and the accumulation mechanism of high levels of amino acids in tea plants [29].

In view of this, different genotypes of tea plant cultivars aged 7 between and 40 years were used as the research materials, and metabolomics and transcriptomics technology were combined to explore the mechanism behind the differences in the metabolites of spring tender shoots in this work. Our main objectives were as follows: (1) to screen the differentially expressed metabolites (DEMs) and differentially expressed genes (DEGs) of tea cultivars of different genotypes at different plant ages and (2) to clarify the regulation mechanism of the main DEMs and DEGs. These findings help to further our understanding of the molecular and metabolic mechanisms of metabolites in the tender shoots of different genotypes of tea plants, as well as providing valuable reference information for the accurate utilization of fresh leaves of tea plants of different ages.

## 2. Materials and Methods

### 2.1. Experimental Materials

The two tea cultivars used in this study were Gancha 4 and Wulv 1. Gancha 4 was bred from the hybrid offspring of Fuding Dabaicha and Huangyezao, while Wulv 1 was bred from the Wuyuan tea population; these represent two different tea cultivar genotypes. The plant ages of the two tea cultivars were 7 years (7-year-old Wulv 1, WL-7 and 7-year-old Gancha 4, GC-7) and 40 years (40-year-old Wulv 1, WL-40 and 40-year-old Gancha 4, GC-40), respectively. The 7-year-old tea plant was propagated by cutting and planted through the cuttings of the 40-year-old tea plant. The four test materials were planted in the same plot of the tea germplasm resource nursery of Jiangxi Cash Crop Research Institute (N 28°22′20″, E 116°0′6″). The planting method used single rows and double planting, with 1.5 m row spacing and 0.3 m plant spacing. Except for plant age, all the other planting management methods (including harvesting, pruning, and fertilization) of the two cultivars were exactly the same as those used in local tea plantations. On 30 March 2021, the phenological period of the experimental materials reached the stage of one bud and two leaves. One bud and two leaves of the four materials were harvested, respectively, from at least 30 tea plants of each cultivar, and were immediately fixed with liquid nitrogen. Each sample included six independent biological replicates at the time of picking. Six biological replicates were used for each sample for metabolomics detection, and three biological replicates were randomly selected from six biological replicates for transcriptomic and biochemical component detection. All the samples were stored in a −80 °C refrigerator.

### 2.2. Experimental Method

#### 2.2.1. Metabolite Detection

The extraction of metabolites was carried out as follows: the freeze-dried crushed samples of 100 ± 0.1 mg were weighed and placed in a 2 mL centrifuge tube, with the addition of 400 μL methanol solution, vortexed for 1 min, extracted in an ultrasonic bath for 30 min, and then centrifuged for 10 min at 12,000 rpm and 4 °C. The supernatant was transferred to a new 2 mL centrifuge tube and then concentrated and dried in a 5305 vacuum concentrator (Eppendorf, Hamburg, Germany), before it was re-dissolved by adding 150 μL of 2-chloro-l-phenylalanine (4 ppm) solution prepared with 80% methanol water (stored at 4 °C). The supernatant was filtered using a 0.22 μm PTFE membrane (Jinteng, Tianjin, China), and the filtrate was added to the flask for liquid chromatography–mass spectrometry (LC–MS) detection. Six biological replicates were performed for each sample.

Quality control sample processing was carried out, with quality control samples (QC) prepared from the same amount of mixed crushed samples of the four materials. They were extracted and detected using the same method as that used for the analytical samples, and the QC samples were repeated six times. In the process of instrument detection, one QC sample was inserted into every ten detection and analysis samples to monitor the repeatability of the whole analysis process.

As for the LC–MS analysis of metabolites, a Thermo Vanquish LC–MS (Thermo Fisher Scientific, Waltham, MA, USA) was used to identify metabolites. The chromatographic column used was an ACQUITY UPLC^®^ HSST3 (2.1 × 150 mm, 1.8 μm) (Waters, Milford, MA, USA). In the negative ion mode, mobile phase A was acetonitrile, and mobile phase B was a 5 mM ammonium formate solution. The gradient elution of mobile phase A was 0–1 min, 2%; 1–9 min, 2–50%; 9–12 min, 50–98%; 12–13.5 min, 98%; 13.5–14 min, 98–2%; 14–17 min, 2%. In the positive ion mode, mobile phase C was a 0.1% formic acid acetonitrile, and mobile phase D was 0.1% formic acid solution. The gradient elution of mobile phase C was 0–1 min, 2%; 1–9 min, 2–50%; 9–12 min, 50–98%; 12–13.5 min, 98%; 13.5–14 min, 98–2%; 14–20 min, 2%. The flow rate was 0.25 mL/min, the column temperature was 40 °C, and the injection volume was 2 μL.

A Thermo Q active focus mass spectrometer detector (Thermo Fisher Scientific, USA) and an electrospray ion source (ESI) were used for mass spectrometry. The data were collected in the positive and negative ion modes. The positive ion spray voltage was 3.50 kV, the negative ion spray voltage was −2.50 kV, the sheath gas was 30 arb, and the auxiliary gas was 10 arb. The capillary temperature was 325 °C. The first-stage full scan was carried out with a resolution of 70,000; the first-stage ion scanning range was 81–1000 *m*/*z*; and the second-stage cleavage was carried out using an HCD. The collision voltage was 30%, and the second-stage resolution was 17,500.

#### 2.2.2. Transcriptome Sequencing

Total RNA was extracted using the TRIzol reagent (Invitrogen, Waltham, MA, USA) according to the manufacturer’s instructions. The total RNA qualified by quality inspection was sent to Beijing Novogene Technology Co., Ltd. (Beijing, China) for cDNA library construction and transcriptome sequencing. A total of 12 libraries (WL-7, WL-40, GC-7, and GC-40 × 3 biological duplicates) were constructed and sequenced using the Illumina platform based on the Paired-End 150 (PE150) strategy. Clean map readings were uploaded to the “Shuchazao” reference genome using the HISAT2 tool with the default parameters [3]. Fragments per kilobase of transcripts per million fragments mapped (FPKM) were performed to calculate gene expression levels. Then, the DEGs were screened using the criteria of log_2_ (fold change) ≥1.5 and a *p*-value of <0.01. Gene ontology (GO) enrichment analysis of the DEGs was carried out using the GOseq R software package, and KOBAS software was used to test the statistical enrichment of the DEGs in the Kyoto Encyclopedia of Genes and Genomes (KEGG) path. The raw sequence data were deposited in the Genome Sequence Archive at the National Genomics Data Center, China National Center for Bioinformation, under accession number CRA007305, which is publicly accessible at https://bigd.big.ac.cn/gsa, accessed on 26 June 2022 [30].

#### 2.2.3. Quantitative Real-Time PCR (qRT-PCR) Analysis

Ten genes related to flavonoids and amino acids were selected for gene expression verification using qRT-PCR. The PrimeScript™ RT reagent kit with gDNA Eraser (TaKaRa, Kusatsu, Shiga) was used to reverse transcribe mRNA into cDNA, and NCBI (http://www.ncbi.nlm.nih.gov/tools/primer-blast/, accessed on 21 March 2022 [31]) was used to design the gene-specific primers. The primers used in this study are listed in Table S1. The qRT-PCR (Yeasen, Shanghai, China) reactions were conducted using the following parameters: 95 °C for 10 min, 45 cycles at 94 °C for 10 s, and 58 °C for 15 s. The glyceraldehyde-3-phosphate dehydrogenase (GAPDH) was used as an internal reference, and the gene expression levels were calculated using the 2−ΔΔCT method.

#### 2.2.4. Biochemical Component Detection

Water extract, tea polyphenols, and free amino acids were detected according to GB/T 8305-2013 (differential method) (GB/T: National Standards of People’s Republic of China) [32], GB/T 8313-2018 (Folin–Ciocalteu phenol colorimetry) [33], and GB/T 8334-2013 (ninhydrin colorimetric method) [34], respectively. Briefly, the extraction method used for the water extract and free amino acids was as follows: we added 300 mL of boiling water to 2.0 ± 0.001 g of crushed freeze-dried sample, and extracted it in a 100 °C water bath for 45 min. The extraction method for the tea polyphenols was as follows: 0.2 ± 0.001 g of crushed freeze-dried sample was placed into a 10 mL centrifuge tube, and then 5 mL of 70% methanol preheated at 70 °C was added. After extraction for 30 min in a 70 °C water bath, the extracting solution was placed a centrifuge. We repeated the extraction with 5 mL of 70% methanol. Three biological replicates were performed for each sample.

### 2.3. Data Statistics and Analysis

ProteoWizard (v3.0.8789) was used to process the original data of the metabolomics. The XCMS package of R (v3.3.2) was used for peak identification, peak filtration, and peak alignment, and the data matrix including the mass-to-charge ratio (*m*/*z*), retention time, and peak area (intensity) was obtained. The metabolites were identified according to the accurate molecular weight, the MS/MS secondary fragment information, and information from the relevant literature regarding tea [10,35]. One-way analysis of variance (ANOVA) with the least significant difference (LSD) was performed using SPSS19.0. The heat map was drawn using Hiplot software (https://hiplot.com.cn, accessed on 8 March 2022) [36]. Principal component analysis (PCA), orthogonal partial least squares discrimination analysis (OPLS-DA), and orthogonal partial least squares analysis (O2PLS) were implemented using SIMCA 14.0.

## 3. Results and Discussion

### 3.1. Biochemical Component Analysis

**Figure 1:**
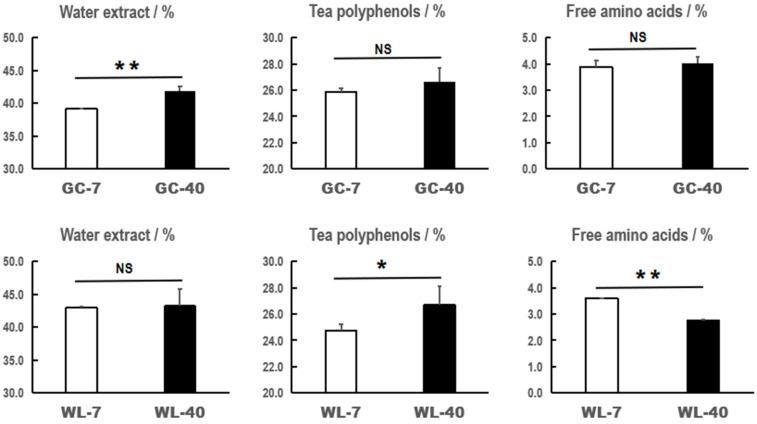
The contents of the biochemical components of two tea cultivars of different plant ages. Here, * is *p* < 0.05 and ** is *p* < 0.01; NS is not significant.

Tea polyphenols, also known as tea tannins, mainly include catechins, flavonoids, anthocyanidins, phenolic acid, and depsides [37]. Free amino acids in tea include protein amino acids and non-protein amino acids [38]. The water extract is the sum of the water-soluble substances in tea [39]. These substances are considered to be important biochemical components affecting the quality of tea [40]. In this study, three main biochemical components of the tender shoots of plants of different ages from two cultivars were detected and analyzed (Figure 1). We found that the effects of plant age on the biochemical components of the two tea cultivars were inconsistent. The water extract content in GC-40 was significantly higher than that of GC-7 (*p* < 0.01), which increased from 39.16% to 41.89%. The content of tea polyphenols and free amino acids in GC-40 was slightly higher than that in GC-7 (not significant). The polyphenol content in WL-40 was significantly higher than in WL-7 (*p* < 0.05), with an increase from 24.75% to 26.69%. The free amino acid content of WL-40 was significantly lower than that of WL-7 (*p* < 0.01), with a decrease from 3.60% to 2.75%. There was no significant difference between their water extract contents. Therefore, the increase in plant age significantly promoted the content of water extract in Gancha 4, but plant age had no significant effect on free amino acid or tea polyphenol contents. For Wulv 1, the increase in plant age significantly induced the content of tea polyphenols and a significantly decreased content of free amino acids, but had no significant effect on its water extract.

### 3.2. Metabonomic Analysis

**Figure 2:**
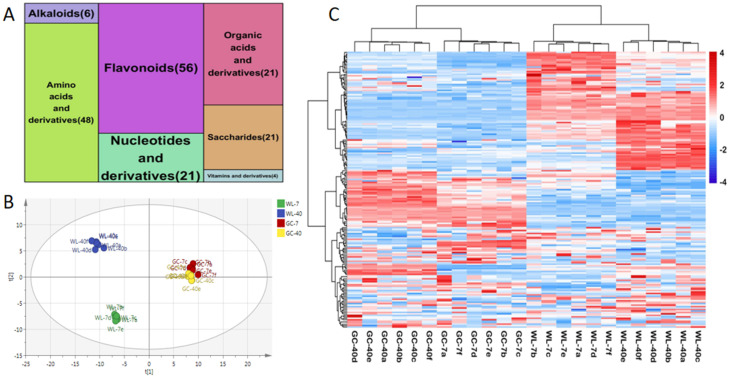
Analysis of the metabolome of plants of different ages from two tea cultivars. (**A**) Tree map of metabolite classification. (**B**) PCA analysis, R^2^X = 0.704, Q^2^ = 0.543. (**C**) Heat map of total metabolites; red indicates a high content, blue indicates a low content, and white represents an average value.

In this study, LC-ESI-MS/MS metabolomics was used to characterize the overall chemical characteristics of the plants of different ages from the two cultivars. The precursor molecules obtained in the positive and negative ion modes were analyzed by PCA. It was found that six QC samples were effectively aggregated, indicating good repeatability (Figure S1). A total of 189 metabolites were identified, which were divided into 7 categories (Figure 2A), including 56 flavonoids, 48 amino acids and their derivatives, 21 saccharides, 33 organic acids and their derivatives, 21 nucleotides and their derivatives, 6 alkaloids, and 4 vitamins and their derivatives (Table S2).

The original state of the metabolite data can be reflected by using PCA analysis [41]. The PCA analysis of the metabolites of the plants of different ages from the two cultivars revealed that WL-40 and WL-7 were far apart in the first two principal component score diagrams, while GC-40 and GC-7 were close (Figure 2B). The results showed that the metabolite difference between WL-40 and WL-7 was large, whereas the difference between GC-40 and GC-7 was relatively small. Through a heat map analysis of all the metabolites identified in the plants of different ages from the two cultivars (Figure 2C), we found that the difference in the metabolite abundance of WL-40 and WL-7 was large (the red area and the blue area of the two samples differ greatly, as shown), but this difference was relatively small in GC-40 and GC-7 (the difference between the red area and the blue area is relatively small, as shown). The results were identical to those of the PCA. Overall, plant age could affect the metabolites of tea tender shoots in spring. It has been reported that there are significant differences in the metabolic profiles of the leaves of 10-, 35-, and 55-year-old Shuixian cultivar plants [20], and there are significant metabolic differences between the fresh tea leaves of the 8- and 25-year-old Yabukita cultivar plants [18]. These conclusions are in agreement with the results of this study. Additionally, this study further revealed that the influence of plant age on tea cultivars of different genotypes differed, and that the influence on the metabolites in Wulv 1 was greater than that in Gancha 4, suggesting that the influence of plant age on tea metabolites is cultivar-specific.

### 3.3. Screening of DEMs

**Figure 3:**
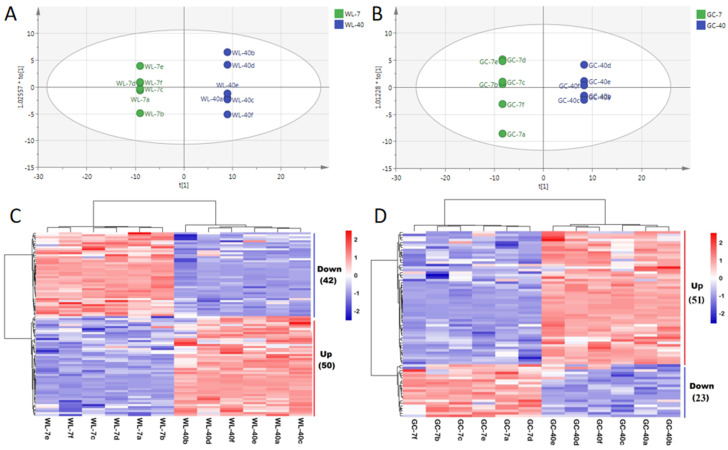
Screening of DEMs of two tea cultivars at different ages. The OPLS-DA of Wulv 1 (**A**) and Gancha 4 (**B**). Heat map of DEMs of Wulv 1 (**C**) and Gancha 4 (**D**). Red indicates high content, blue indicates low content, and white represents average value.

Here, we employed OPLS-DA, as it is an effective method for screening DEMs [10]. In order to screen the DEMs of tea plants of different ages from the two cultivars, OPLS-DA analysis was performed with all of the relevant metabolites as variables. The results demonstrated that the two OPLS-DA models established could effectively distinguish the samples of plants of different ages from the two cultivars (Figure 3A,B). Cross-validation indicated that the interpretation degree of the two models was high, and there was no fitting phenomenon (Figure S2). Their DEMs were screened according to the principles of VIP > 1.00 and *p* < 0.05 [10] (Table S3). There were 92 DEMs shared between WL-7 and WL-40, among which 50 metabolites were upregulated and 42 metabolites were downregulated in WL-40 (Figure 3C). The 50 upregulated metabolites included 21 flavonoids, 6 amino acids and their derivatives, 7 saccharides, 9 organic acids and their derivatives, and 7 nucleotides and their derivatives. There were 2 flavonoids, 25 amino acids and their derivatives, 8 organic acids and their derivatives, 1 saccharide, and 6 nucleotides and their derivatives in the 42 downregulated metabolites. There were 74 DEMs shared between GC-7 and GC-40, among which 51 metabolites were upregulated and 23 metabolites were downregulated in GC-40 (Figure 3D). Among the 51 upregulated metabolites, 13 flavonoids, 21 amino acids and their derivatives, 3 saccharides, 8 organic acids and their derivatives, and 6 nucleotides and their derivatives were identified. There were 6 flavonoids, 9 amino acids and their derivatives, 3 organic acids and their derivatives, 3 saccharides, and 2 nucleotides and their derivatives in the 23 downregulated metabolisms.

According to the results of the DEMs of plants of different ages from the two cultivars, in contrast to the 7-year-old tea plants, the upregulated metabolites in the 40-year-old tea plants were more numerous than the downregulated metabolites, among which flavonoids and amino acids and their derivatives were predominant. These two kinds of metabolites are also considered to have a greater impact on the quality of tea [10]. Wang. [20] reached the same conclusion when studying the DEMs in the leaves of plants of different ages from the Shuixian cultivars, that is, plant age showed the greatest regulation of the amino acid and phenolic metabolites of the tea, and the upregulated metabolites were more numerous than the downregulated metabolites in the old plants. Furthermore, we also observed that amino acids and their derivatives were the most numerous in the downregulated metabolites of both cultivars. The difference was that, among the upregulated metabolites, the most numerous in Wulv 1 were flavonoids, while the most numerous in Gancha 4 were amino acids and their derivatives (amino acid derivatives, such as methylation and acetylation), followed by flavonoids. In tea plants, L-theanine, L-aspartic acid, and L-glutamic acid are three kinds of free amino acids found at high levels. Among them, L-theanine has been found to account for more than 60% of the total free amino acids in the tender shoots of tea plants [38]. However, L-glutamic acid was not a differential metabolite in the two tea cultivars. Here, L-theanine and L-aspartic acid were found to belong to DEMs. The expression of these two amino acids was inconsistent in plants of different ages from the two tea cultivars. They were downregulated metabolites in WL-40 and upregulated metabolites in GC-40 (Table S3). Based on the results of the biochemical and metabolic components, we determined that the reason for the high content of water extract in 40-year-old tea plants was that the total upregulated DEMs were more numerous than downregulated DEMs. The high content of tea polyphenols in the 40-year-old tea plants was due to the upregulated expression of abundant flavonoids. The reason why the content of free amino acids in plants of different ages from the two cultivars changed inconsistently was that the number of downregulated amino acids and their derivatives in WL-40 was greater than that in upregulated ones, especially in terms of the downregulation of L-theanine and L-aspartic acid. However, the number of amino acids and their derivatives upregulated in GC-40 was found to be greater than that of the downregulated ones, especially in terms of the upregulation of L-theanine and L-aspartic acid.

Flavonoids belong to phenols, which are mainly bitter and astringent, while free amino acids are mainly umami and sweet [10]. Therefore, within a moderate range, a low content of flavonoids and other phenolic compounds and a high content of free amino acids in tea tender shoots are the material basis for making high-quality tea [14]. According to the results of the biochemical components analysis and total metabolic spectrum, GC-40 was found to still have the material basis for making high-quality tea because the contents of flavonoids and free amino acids were upregulated. However, Wulv 1 was different in that the flavonoids in 40-year-old plants were significantly upregulated, while the content of free amino acids was significantly downregulated. Therefore, in contrast to WL-7, WL-40 has a poorer material basis for making high-quality tea. Nonetheless, due to the significant upregulation of tea polyphenols, WL-40 was more suitable for the development and use of healthy functional products. In general, the genotypes of tea plants play a decisive role in whether old tea plants can produce high-quality tea.

### 3.4. Transcriptomics and DEGs Enrichment Analysis

In order to further explore the molecular mechanism behind the influence of plant age on the metabolic differences between the two tea cultivars, transcriptome sequencing analysis was performed on four materials. A total of 12 cDNA libraries were constructed and sequenced, and 77.69 Gb of clean data were obtained. The clean data of each sample reached 6.10 Gb, and the percentage of the Q30 nucleobase was 93.08% or above, illustrating the high quality of the transcriptomic data. Clean reads of each sample were sequentially compared with the reference genome of “Shuchazao”, and the comparison efficiency was 82.91–84.84% (Table S4). Then, PCA was performed based on the FPKM value of each gene expression in the samples. The four samples showed good repeatability in the PCA, and there were significant differences between GC-40 and GC-7, as well as between WL-40 and WL-7; different years of the same cultivars were distinguished from each other (Figure S3), indicating that gene expression was significantly different between the two cultivars at different plant ages.

**Figure 4:**
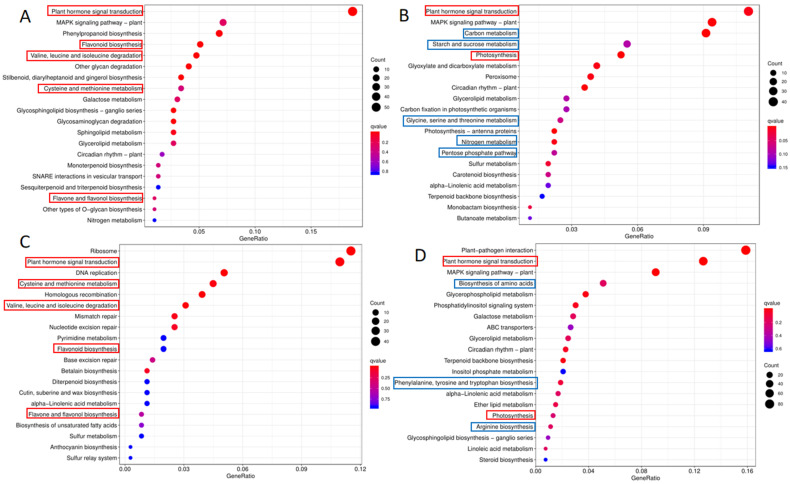
The KEGG enrichment analysis of DEGs. KEGG enrichment analysis of upregulated (**A**) and downregulated (**B**) DEGs in Wulv 1 plants of different ages. The KEGG enrichment analysis of upregulation (**C**) and downregulation (**D**) DEGs of Gancha 4 plants of different ages.

A total of 1717 DEGs were screened in WL-40 and WL-7, of which 770 were upregulated and 947 were downregulated. A total of 2484 DEGs were screened in GC-40 and GC-7, of which 1069 were upregulated and 1415 were downregulated (Figure S4). The GO enrichment analysis of the DEGs revealed that metabolic processes and cellular processes were the most abundant terms in the biological process, and catalytic activity and binding were the most abundant terms in the molecular functions of the two tea cultivars (Figure S5). These terms, mainly annotated by DEGs, were closely related to the metabolic regulation of tea plants, suggesting that the differences in the metabolites of tea plants of different ages were regulated by transcription. We conducted KEGG enrichment analysis for the upregulated and downregulated DEGs of tea plants of different ages from the two tea cultivars. The top 20 pathways with the highest enrichment degree are shown in Figure 4. The upregulated DEGs of Wulv 1 and Gancha 4 were more concentrated, while the downregulated ones were more diversified. The upregulated DEGs were mainly enriched in the pathways common to the two cultivars, such as plant hormone signal transduction, flavonoid biosynthesis, valine, leucine and isoleucine degradation, cysteine and methionine metabolism, and flavone and flavonol biosynthesis (Figure 4A,C). These pathways are bound up with flavonoid biosynthesis, amino acid degradation, and the biosynthesis of tea plants [26,29]. The main common enriched pathways of downregulated DEGs were plant hormone signal transduction and photosynthesis (Figure 4B,D). The related genes in the photosynthesis pathway were downregulated, indicating that the photosynthesis of the two tea cultivars at the age of 40 was lower than that at the age of 7. In the downregulated DEG enrichment pathway of Wulv 1, carbon metabolism, starch and sucrose metabolism, carbon fixation in photosynthetic organizations, and the pentose phosphate pathway were all related to photosynthesis and respiration (Figure 4B) [42]. However, these pathways were not enriched in Gancha 4 (Figure 4D). This illustrates that, in comparison to WL-7, the functions of photosynthesis and carbon assimilation of WL-40 decreased to a greater extent, while in GC-40, these decreased by a relatively smaller extent. The downregulated DEGs of GC-40 were massively enriched in the biosynthesis of amino acids, phenylalanine, tyrosine and tryptophan biosynthesis, and arginine biosynthesis (Figure 4D). These pathways are mainly involved in the amino acid metabolism [29].

To verify the reliability of the transcriptome data, 10 genes in the flavonoid and amino acid biosynthetic pathways were selected for qPCR analysis. All of them showed concordant expression patterns between the transcriptome data and the qPCR results (Figure S6). This result suggests the reproducibility and reliability of the transcriptome expression data.

### 3.5. Metabolic Pathway Changes in Flavonoids and Amino Acids

Based on the identification of DEMs and the enrichment results of the DEGs, we found that plant age had a great impact on the metabolism of flavonoids and amino acids in different tea cultivars. These two substances were important contributors to the flavor and function of the tea. Therefore, we focused on the analysis and exploration of the expression patterns of DEGs related to the metabolic pathways of these two kinds of metabolites.

#### 3.5.1. Flavonoid Metabolic Pathway

**Figure 5:**
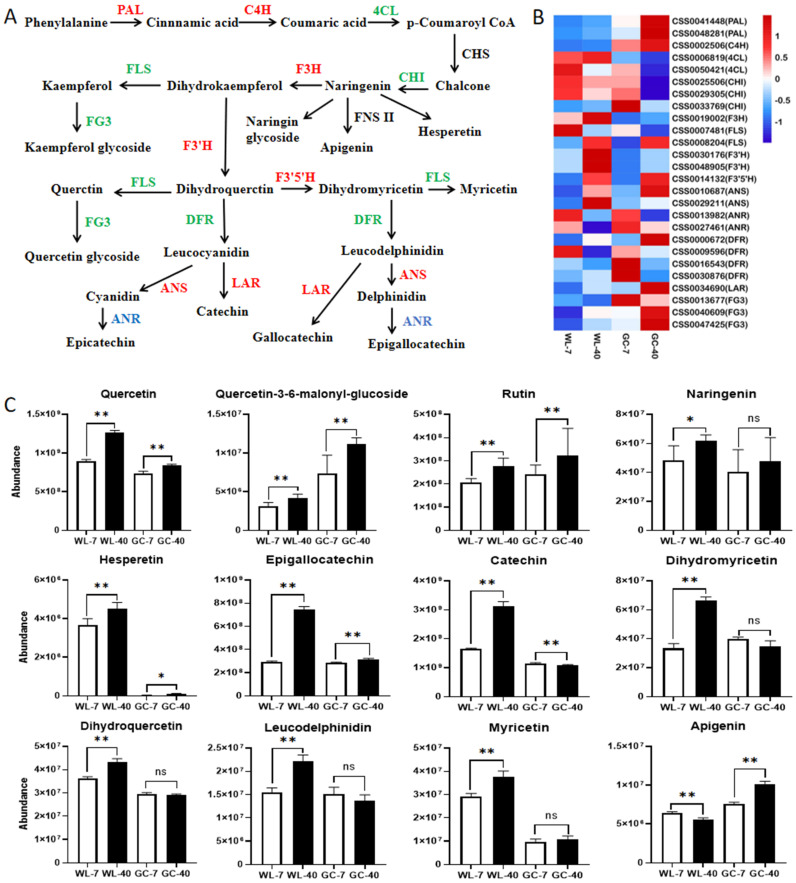
Effect of plant age on the flavonoid metabolism of tea plants. (**A**) Simplified pathway of flavonoid metabolism; red represents upregulation, blue represents downregulation, and green represents both upregulation and downregulation. (**B**) Heat map of key DEGs; low, medium, and high expression levels of the genes are shown in blue, white, and red, respectively. (**C**) Changes in the main differential flavonoids. * is *p* < 0.05 and ** is *p* < 0.01; ns is not significant. Abbreviations are defined as follows: PAL, phenylalanine ammonia-lyase; C4H, cinnamate 4-hydroxylase; 4CL, 4-coumarate–CoA ligase; CHS, chalcone synthase; CHI, chalcone isomerase; F3H, flavanone 3-hydroxylase; FLS, flavonol synthase; F3′5′H, flavonoid 3′,5′-hydroxylase; F3′H, flavonoid 3′-monooxygenase; DFR, dihydroflavonol-4-reductase; ANS, anthocyanidin synthase; ANR, anthocyanidin reductase; FNS II: flavone synthase II; FG3, flavonol-3-O-glucoside galactoside glucosyltransferase; LAR, leucoanthocyanidin reductase.

In the flavonoid biosynthesis pathway (Figure 5A), a total of 26 DEGs were identified, among which 14 DEGs were upregulated in both cultivars, 9 DEGs were downregulated, and 3 DEGs were inconsistent in both cultivars (Figure 5B). The biosynthesis of flavonoids begins with phenylalanine, which is catalyzed by phenylpropanoid-related core biosynthesis genes including phenylalanine ammonia-lyase (PAL), cinnamate 4-hydroxylase gene (C4H), and 4-coumarate-coa ligase gene (4CL). This provides precursors for the biosynthesis of all the major secondary phenolic metabolites in tea plants [43]. The three DEGs encoding PAL and C4H were upregulated in the 40-year-old tea plants of the two cultivars, which might promote an overall increase in flavonoid abundance in 40-year-old tea plants. Chalcone synthase (CHS) catalyzes the biosynthesis of chalcone, and chalcone isomerase (CHI) catalyzes the isomerization of chalcone into naringenin [44]. Naringenin is the first stable intermediate product in the flavonoid biosynthesis pathway, and it and chalcone are both important intermediate products from which other flavonoids are synthesized [44]. The gene encoding CHS was not differentially expressed in the plants of different ages from the two cultivars, and chalcone was not a differential metabolite. The expression of the genes encoding 4CL (CSS0006819) and CHI (CSS0006819) was inconsistent in the plants of different ages from the two cultivars. The expression of these two genes was upregulated in WL-40 and downregulated in GC-40. Among the differential metabolites, the abundance of naringenin increased significantly in WL-40, but not in GC-40 (Figure 5C). The variation trend of gene expression and metabolites was consistent. As naringenin was the first stable intermediate in the flavonoid biosynthesis pathway, it might have had an effect on the significant increase in tea polyphenols in WL-40, but not in GC-40. Flavonol synthase (FLS) catalyzes the biosynthesis of flavonol. The CSS0008204 of the gene encoding FLS was upregulated in the both cultivars, and the abundance of some flavonols and their glycosides (quercetin, quercetin-3–6-malonyl-glucoside, rutin, and myricetin) increased in the 40-year-old tea plants (Figure 5C). Flavonoid 3′,5′-hydroxylase (F3′5′H) and flavonoid 3′-monooxygenase (F3′H) catalyze the biosynthesis of dihydromyricetin and dihydroquercetin, respectively [42]. Both genes encoding these two enzymes were upregulated in the 40-year-old tea plants. The abundance of dihydromyricetin and dihydroquercetin increased in WL-40, but the abundance showed no significant difference in GC-40. Anthocyanidin synthase (ANS) catalyzes the biosynthesis of anthocyanidins. The genes encoding ANS were upregulated in the 40-year-old tea plants, and the abundance of delphinidin-3,7-di-O-beta-D-glucoside was increased in GC-40. The abundance of delphinidin-3-rutinoside increased in WL-40 (Figure 5C). Leucoanthocyanidin reductase (LAR) catalyzes the biosynthesis of catechin. The genes encoding LAR were upregulated in the 40-year-old tea plants, and the abundance of catechin was increased in WL-40 and decreased in GC-40. Catechins can further form anthocyanins or phenotypic catechins under the catalysis of leucoanthocyanidin dioxygenase (LDOX), but the biosynthetic mechanism is not clear [45]. This may be the reason for the differences between the two cultivars, and additional experiments need to be designed for further exploration. Dihydroflavonol-4-reductase (DFR) catalyzes the biosynthesis of leucoanthocyanidins. Four differential genes encoding DFR were identified, three of which were downregulated, while one was upregulated. The abundance of leucodelphinidin in WL-40 was increased, implying that the upregulated gene (CSS0000672) had a dominant role among the four differential genes. The abundance of epigallocatechin (EGC) increased in the 40-year-old tea plants of both cultivars. Epicatechin (EC) increased in WL-40, but there was no significant difference in EC in GC-40. Anthocyanidin reductase (ANR) is involved in the biosynthesis of the two catechins. The expression of two different genes encoding ANR was downregulated in WL-40 and GC-40, suggesting that the expression of different genes was inconsistent with the changes in the abundance of the two catechins. Since EGC and EC are not the terminal products of the metabolic pathway, there may be more complex change mechanisms that promote this phenomenon, and these need to be further studied.

On the whole, in comparison to the 7-year-old tea plants, the upregulated expression of key genes in the flavonoid metabolism pathway in the 40-year-old tea plants was higher than that of the downregulated expression. These upregulated genes were found to promote the biosynthesis of some flavonoids and to increase the abundance of flavonoids in the 40-year-old tea plants. Wang [20] studied the DEGs of plants of the Shuixian cultivar at different ages and similar conclusions were drawn, with the study finding that the changes in the flavonoids were regulated by key genes. In addition, we found that the flavonoid content of tea cultivars with different genotypes was affected differently by plant age, and the flavonoid abundance significantly increased in WL-40 (21 kinds of flavonoids), which was more than in GC-40 (13 kinds of flavonoids). This might also be the main reason why the content of tea polyphenols in WL-40 was significantly increased (*p* < 0.05), while the increase in GC-40 was not significant (*p* > 0.05) in the biochemical components analysis.

#### 3.5.2. Amino Acid Metabolic Pathway

**Figure 6:**
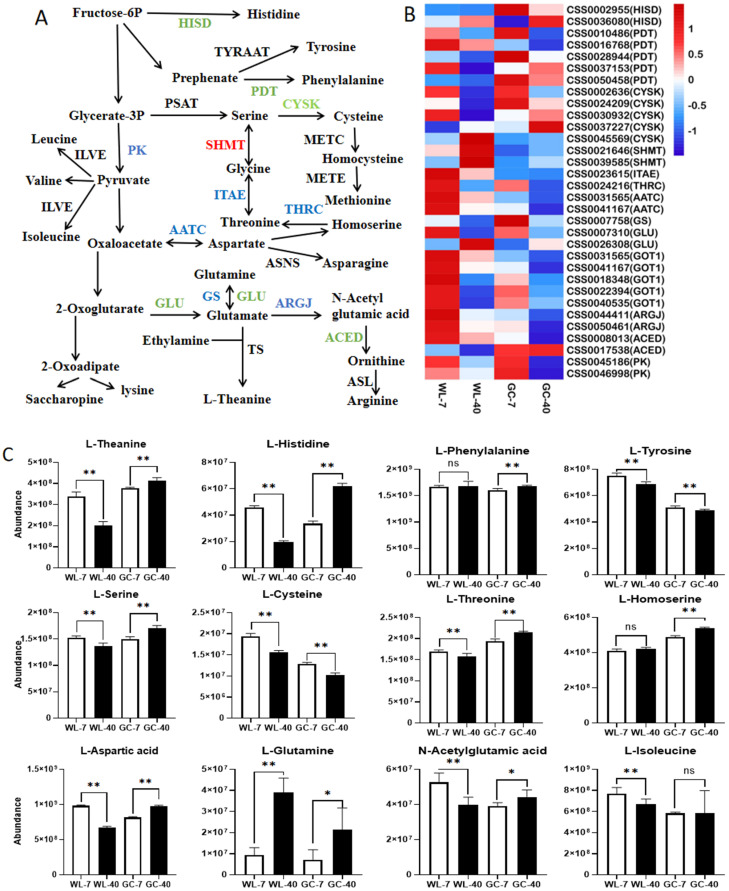
Effects of plant age on amino acid metabolism of tea plants. (**A**) Simplified pathway of amino acid metabolism; red represents upregulation, blue represents downregulation, and green represents both upregulation and downregulation. (**B**) Heat map of key DEGs; low, medium, and high expression levels of genes are shown in blue, white, and red, respectively. (**C**) Changes in the main differentially expressed amino acids. * is *p* < 0.05 and ** is *p* < 0.01; ns is not significant. Abbreviations are defined as follows: HISD, histidinol dehydrogenase; TYRAAT, plant arogenate dehydrogenase; PDT, prephenate dehydratase; PSAT, phosphoserine aminotransferase; CYSK, cysteine synthase; PK, pyruvate kinase; ILVE, branched-chain amino acid aminotransferase; SHMT, serine hydroxymethyltransferase; ITAE, threonine aldolase; METC, cysteine-S-conjugate beta-lyase; METE, homocysteine methyltransferase; THRC, threonine synthase; AATC, aspartate aminotransferase cytoplasmic; ASNS, asparagine synthase; GLU, glutamate synthase; GS, glutamine synthase; ARGJ, glutamate N-acetyltransferase; AECD, acetylornithine deacetylase; ASL, argininosuccinate lyase; TS, theanine synthetase.

In the free amino acid biosynthesis pathway (Figure 6A), a total of 32 DEGs were identified, of which 23 were downregulated in both cultivars, 6 were upregulated, and 3 were inconsistent (Figure 6B). In comparison to WL-7, 23 amino acids and their derivatives were downregulated and 7 were upregulated in WL-40. Downregulated amino acids included L-histidine, L-cysteine, L-threonine, L-aspartic acid, and N-acetylglutamic acid (Figure 6C). The genes involved in encoding the key enzymes of amino acid biosynthesis were also downregulated (CSS0002955, CSS0002636, CSS0024209, CSS0030932, CSS0023615, CSS0031565, CSS0041167, CSS0044411, and CSS0050461) (Figure 6B), indicating that the expression of these DEGs was consistent with the change trend of the corresponding amino acids. This conclusion was consistent with Wang’s results [20], who noted that the changes in the amino acids of tea plants of different ages were regulated by key functional genes in their synthetic pathway. In contrast to Wulv 1, 23 amino acids and their derivatives were upregulated and 7 were downregulated in Gancha 4. There were 26 downregulated DEGs and 6 upregulated DEGs in the amino acid biosynthesis pathway, and the variation trend of the amino acids and their derivative abundance was inconsistent with the trend of gene expression. For example, L-threonine, L-aspartic acid, L-phenylalanine, L-histidine, and N-acetylglutamic acid were upregulated in GC-40 (Figure 6C). However, the genes encoding these amino acid synthetases were downregulated (Figure 6B), and the mechanism of this difference needs to be studied further. The abundance of L-theanine decreased by 40% in WL-40 and increased by 9% in GC-40 (Figure 6C). Theanine synthetase (TS) catalyzes theanine biosynthesis from glutamate and ethylamine [38], and no DEGs involved in encoding TS were identified in the transcriptome in this work. A possible reason for this is that L-theanine was mainly synthesized in the root of the tea plant and then transported to the tender shoots of the tea plant over a long distance [46], while the sample treated in this study consisted of new tender shoots. Furthermore, L-theanine was downregulated in WL-40, but upregulated in GC-40, which might be related to the photosynthesis of tea plants. Previous studies have shown that theanine biosynthesis requires not only a sufficient nitrogen supply, but also a sufficient carbon source and energy, which mainly come from photosynthesis and respiration [47]. Mozumder et al. [18] indicate that the photosynthesis rate of old tea plants is low and that their carbohydrate metabolism is limited, which might reduce the biosynthesis of some metabolites. In the KEGG enrichment analysis, we found that photosynthesis and carbon assimilation were more decreased in WL-40 as compared with WL-7, but that GC-40 showed a relatively smaller decrease (Figure 5A,B). This might be the main reason why the L-theanine content decreased significantly in WL-40, but not in GC-40. The change trend of L-threonine, L-aspartic acid, L-histidine, N-acetylglutamic acid, and L-serine was the same as that of L-theanine, which may also be related to the different effects of plant age on the photosynthesis of different tea cultivars. However, we found no direct evidence to confirm this speculation in this study. Therefore, additional experiments are needed for further exploration.

Hence, through a comprehensive analysis, we found that the downregulated expression of the key genes of amino acid biosynthesis in WL-40 decreased the abundance of amino acids and their derivatives. However, the expression of key genes of amino acid biosynthesis in GC-40 was inconsistent with the change trend of amino acid and their derivative abundance, suggesting that the influence of plant age on amino acids might be cultivar-specific. The difference in L-theanine abundance in the two cultivars at different plant ages might be related to the different degrees of photosynthesis decline in the 40-year-old plants of the two cultivars.

### 3.6. Screening of Key Structural Genes for Flavonoid Biosynthesis

**Figure 7:**
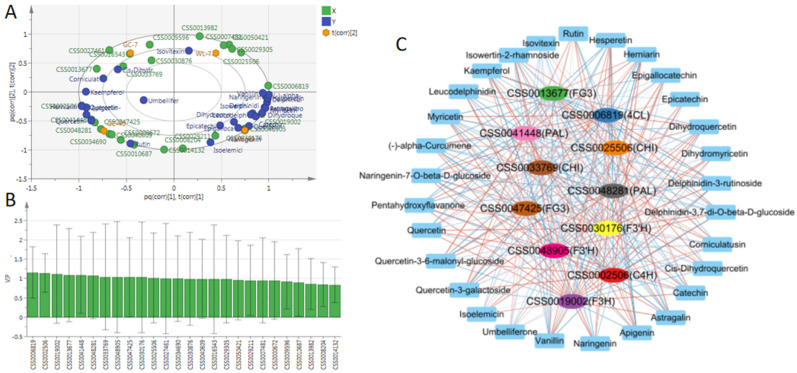
Screening of key genes for flavonoid biosynthesis. (**A**) Load diagram of the O2PLS model. Here, x is the differential gene, y is the differential flavonoid, and t is the sample; R^2^X = 0.898, R^2^Y = 0.845, Q^2^ = 0.305. (**B**) The VIP value of O2PLS model. (**C**) Correlation network of the key regulatory genes and differential flavonoids. Red lines indicate a positive correlation, and blue lines indicate negative correlation.

We found that the upregulation of flavonoid content in the aged tea plants was regulated by the DEGs in their biosynthesis pathways, and that the regulation mechanism of amino acid changes may be cultivar-specific, which needs to be further explored. Therefore, we further identified the key structural genes of flavonoid content differences in plants of different ages. The O2PLS model was established with the above-screened differential flavonoids as the Y variable and the differential genes in the biosynthetic pathway of flavonoids as the X variable (Figure 7A). Then, VIP > 1 and *p* < 0.05 were used as the criteria to screen the key regulatory genes [10]. We found that the VIP values of 11 DEGs were greater than 1 (Figure 7B), so these genes were the key structural genes influencing the differences in flavonoid content in the plants of different ages. The constructed correlation network is shown in Figure 7C. We found that there was a complex correlation between these genes and flavonoid metabolites. The 11 key structural genes include 2 PAL genes, 1 C4H gene, 1 4CL gene, 2 CHI genes, 1 F3H gene, 2 F3′H genes, and 2 FG3 genes. As PAL, C4H, 4CL, and CHI are the key enzymes in the upstream of flavonoid biosynthesis pathway, they play a decisive role in flavonoid biosynthesis [48]. Furthermore, F3H and F3′H catalyze the biosynthesis of dihydrokaempferol and dihydroquercetin, respectively. These two substances are the biosynthetic precursors of anthocyanins, catechins, and flavonoids and their glycosides [49]. It is understood that FG3 catalyzes the synthesis of various flavonoid glycosides from flavonoids [50]. Therefore, the differential expression of these 11 key structural genes in tea plants of different ages may be an important reason for the difference in their flavonoid contents.

## 4. Conclusions

In conclusion, plant age can significantly change the metabolites in the spring tender shoots of tea plants. Flavonoids and amino acids and their derivatives were predominant among the differential metabolites. The contents of most flavonoids in the aged tea plants (40-year-old) of different genotypes were upregulated, which was caused by the upregulated expression of key genes in the flavonoid biosynthesis pathway. We further found that 11 key structural genes played a key regulatory role in the changes in the flavonoid contents of tea plants of different plant ages, and we constructed a correlation network between them. However, amino acids and their derivatives showed different mechanisms. The differences in amino acids and their derivatives in Wulv 1 were regulated by key genes in the biosynthesis pathway; however, in Gancha 4, their differential biosynthesis was inconsistent with the expression of related genes, suggesting that the influence of plant age on amino acids and their derivatives might be cultivar-specific. The change in L-theanine content might be caused by the different effects of plant age on the photosynthesis of different tea cultivars. By characterizing and evaluating all the metabolites of two tea plant cultivars with different genotypes and using plants of different ages, we found that whether an old (40-year-old) tea plant could produce high-quality tea was related to the genotype of the tea plant. For example, 40-year-old Gancha 4 could still be used to process high-quality tea, but 40-year-old Wulv 1 was more suitable for the development and use of functional health products due to the up-regulation of flavonoids and the downregulation of free amino acids.

## Data Availability

The data presented in this study are available within the manuscript and the Supplementary Materials (Tables S1–S4, Figures S1–S6).

## Supplementary Materials

The following supporting information can be downloaded at: https://www.mdpi.com/article/10.3390/foods11152303/s1, Figure S1: Principal component analysis in positive (A) and negative (B) modes; Figure S2: Cross validation of OPLS-DA for Wulv1 (A) and Gancha 4 (B); Figure S3: PCA analysis of transcriptomics; Figure S4: Differentially expressed genes of two cultivars with different plant ages; Figure S5: GO enrichment analysis of differentially expressed genes in Wulv1 (A) and Gancha4 (B) of different plant ages; Figure S6: qPCR validation of transcriptome data. (A) Transcriptome gene expression. (B) qPCR gene expression; Table S1: The primers used in this study; Table S2: Metabolite identification results; Table S3: Screening results of differential metabolites; Table S4: Transcriptome quality.
