# Supplementary material for: Untargeted Metabolomics and Transcriptomics Reveal the Mechanism of Metabolite Differences in Spring Tender Shoots of Tea Plants of Different Ages
Source: Foods. 2022 Aug 2;11(15):2303. doi: 10.3390/foods11152303 (PMC9368032; doi:10.3390/foods11152303)
Supplement: Supplementary file 1 [file foods-11-02303-s001.zip › Support information.pdf]

Table S1 The primers used in this study

Gene ID	Forward primer (5'-3')	Reverse primer
CSS0041448(PAL)	TGCAATCTGAGGTTCTCCCG	CCGACCGACCCCAGAAATAG
CSS0007481(FLS)	CTTACGAGGGGTACGGAAGC	CATCGGCCACTTCTTCTGGT
CSS0030176(F3'H)	TCTCTCTCCCTCGAATGGCA	CTAAGCTCATCCCGGCACAT
CSS0014132(F3'5'H)	ATGCATGTGAGGTGAACGGT	TCCGGCACACATTCTTCTCC
CSS0010687(ANS)	ATTCCGGCAACAAGCGAGTA	GAGCTCTGGTTGAGGGCATT
CSS0000672(DFR)	ATTGTTCGTGCAACCGTTCG	GGCCTCATCAAAGCTCCCTT
CSS0047425(FG3)	TTTTTGCCGCCCTAAAACCG	TCCAACTTCCAAGTCTCCGC
CSS0010486(PDT)	ACAAAACGCACAGAATGCCC	CTTTGGCACTGGAAAACCCG
CSS0007310(GLU)	ACAAAACGCACAGAATGCCC	CTTTGGCACTGGAAAACCCG
CSS0008013(ACED)	AGAGTGGCTGGAATTTGCGA	GGATTCCCTCGGCTGGAAAA

Table S4 transcriptome quality

Samples	Clean reads	Clean bases	%≥Q30	Mapped Reads
WL-7a	21,375,660	6,412,698,000	93.57%	35,879,790 (83.93%)
WL-7b	23,821,013	7,146,303,900	93.51%	39,877,323 (83.70%)
WL-7c	22,251,929	6,675,578,700	93.46%	37,197,856 (83.58%)
WL-50a	21,145,793	6,343,737,900	93.58%	35,658,203 (84.32%)
WL-50b	21,308,708	6,392,612,400	93.51%	35,966,090 (84.39%)
WL-50c	21,249,726	6,374,917,800	93.48%	35,672,234 (83.94%)
GC-7a	21,948,162	6,584,448,600	93.42%	36,396,645 (82.92%)
GC-7b	21,392,207	6,417,662,100	93.31%	35,988,819 (84.12%)
GC-7c	20,320,525	6,096,157,500	93.60%	33,693,547 (82.91%)
GC-50a	21,024,858	6,307,457,400	93.49%	35,673,125 (84.84%)
GC-50b	21,286,406	6,385,921,800	93.59%	36,054,987 (84.69%)
GC-50c	21,849,876	6,554,962,800	93.08%	36,814,023 (84.24%)

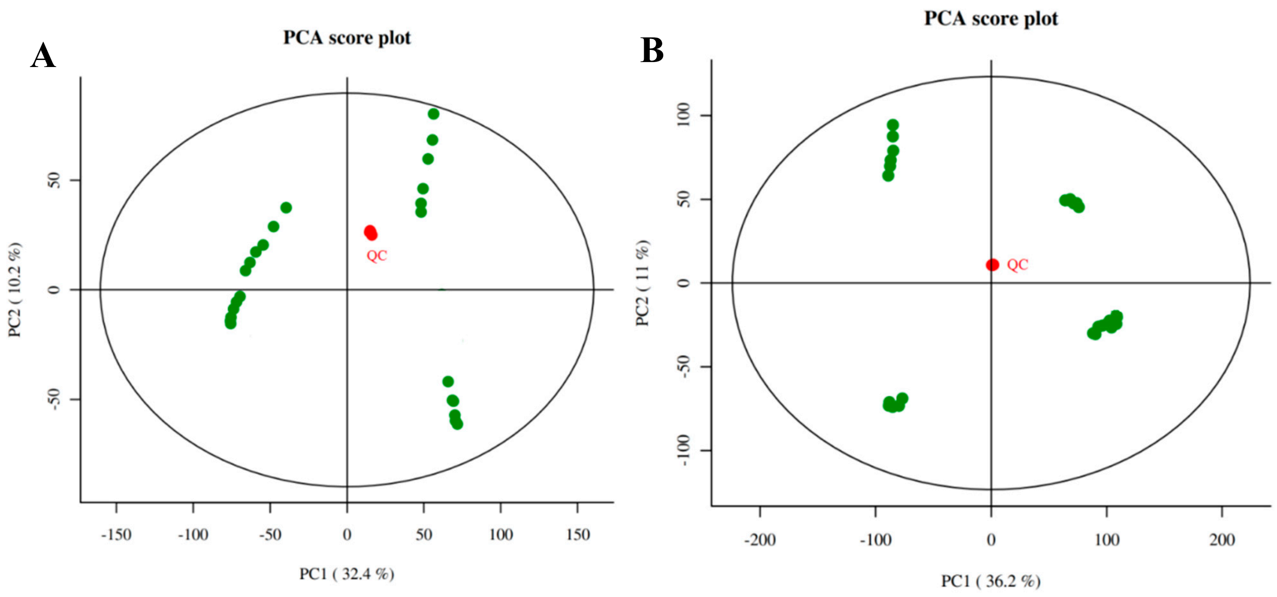

Figure S1 Principal component analysis in positive (A) and negative (B) modes

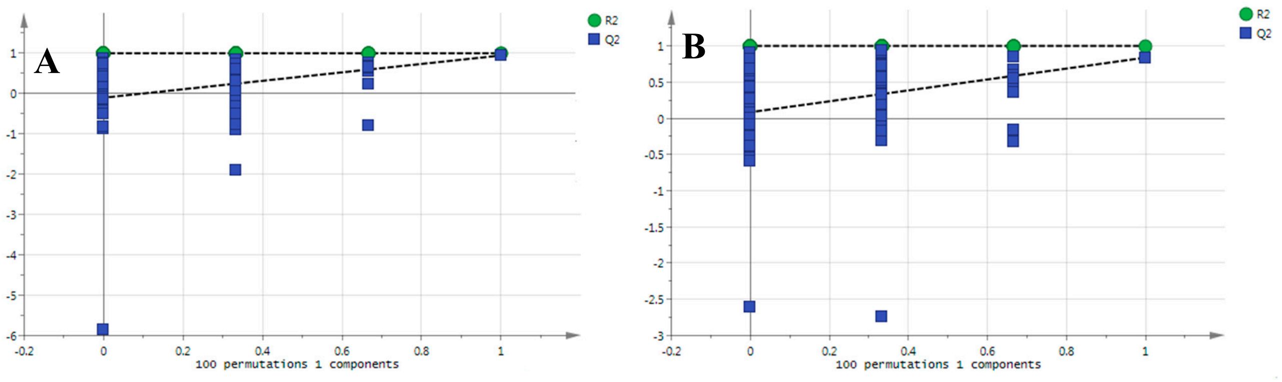

Figure S2 Cross validation of OPLS-DA for Wulv1 (A) and Gancha 4 (B)

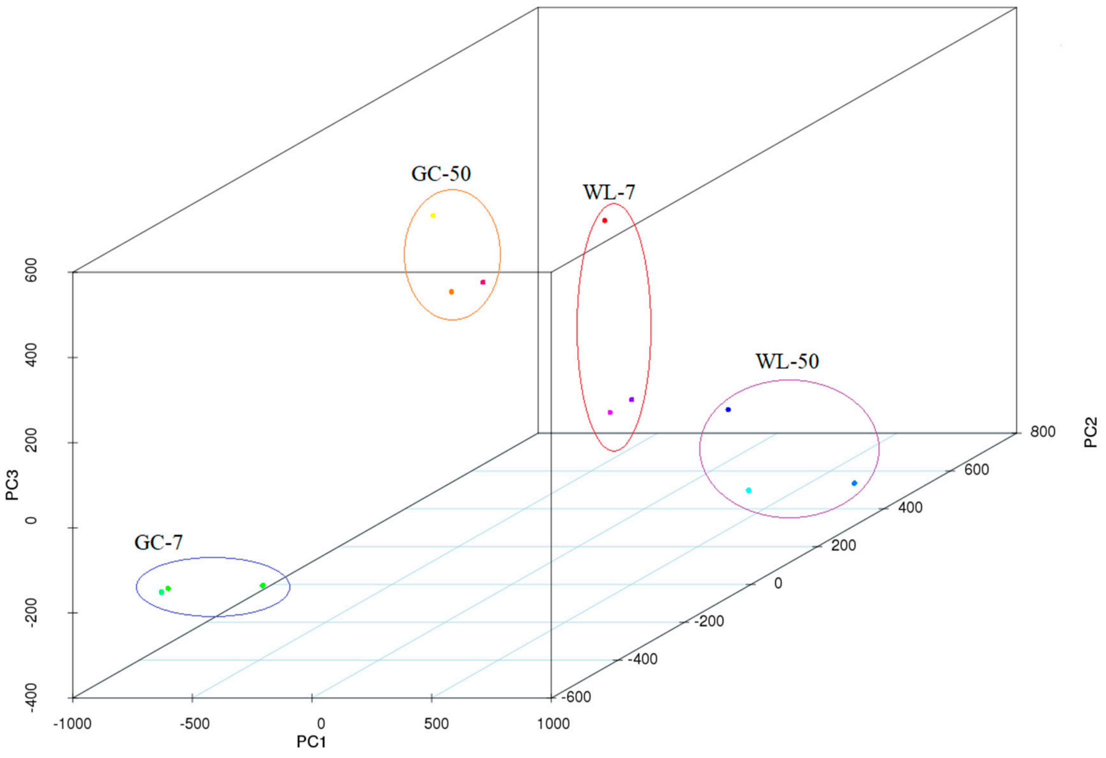

Figure S3 PCA analysis of transcriptomics

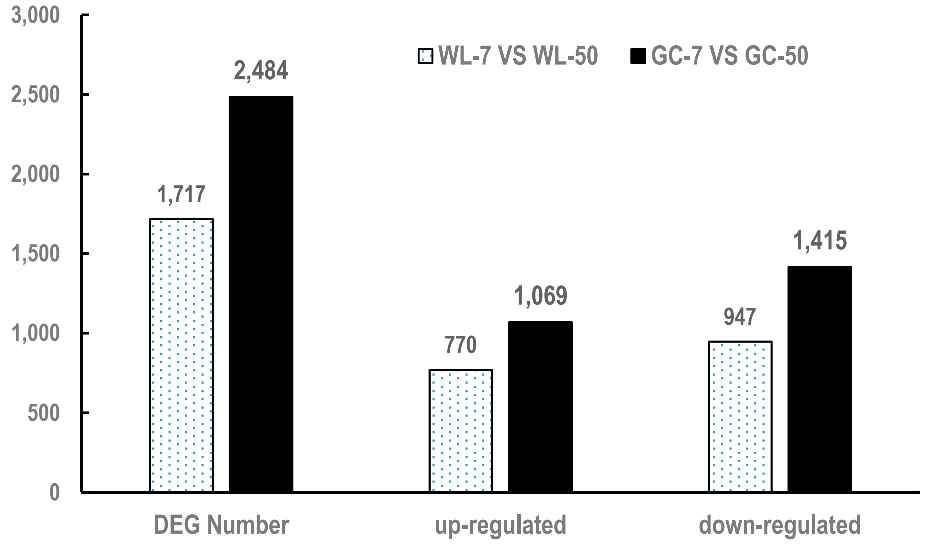

Figure S4. Differentially expressed genes of two cultivars with different plant ages

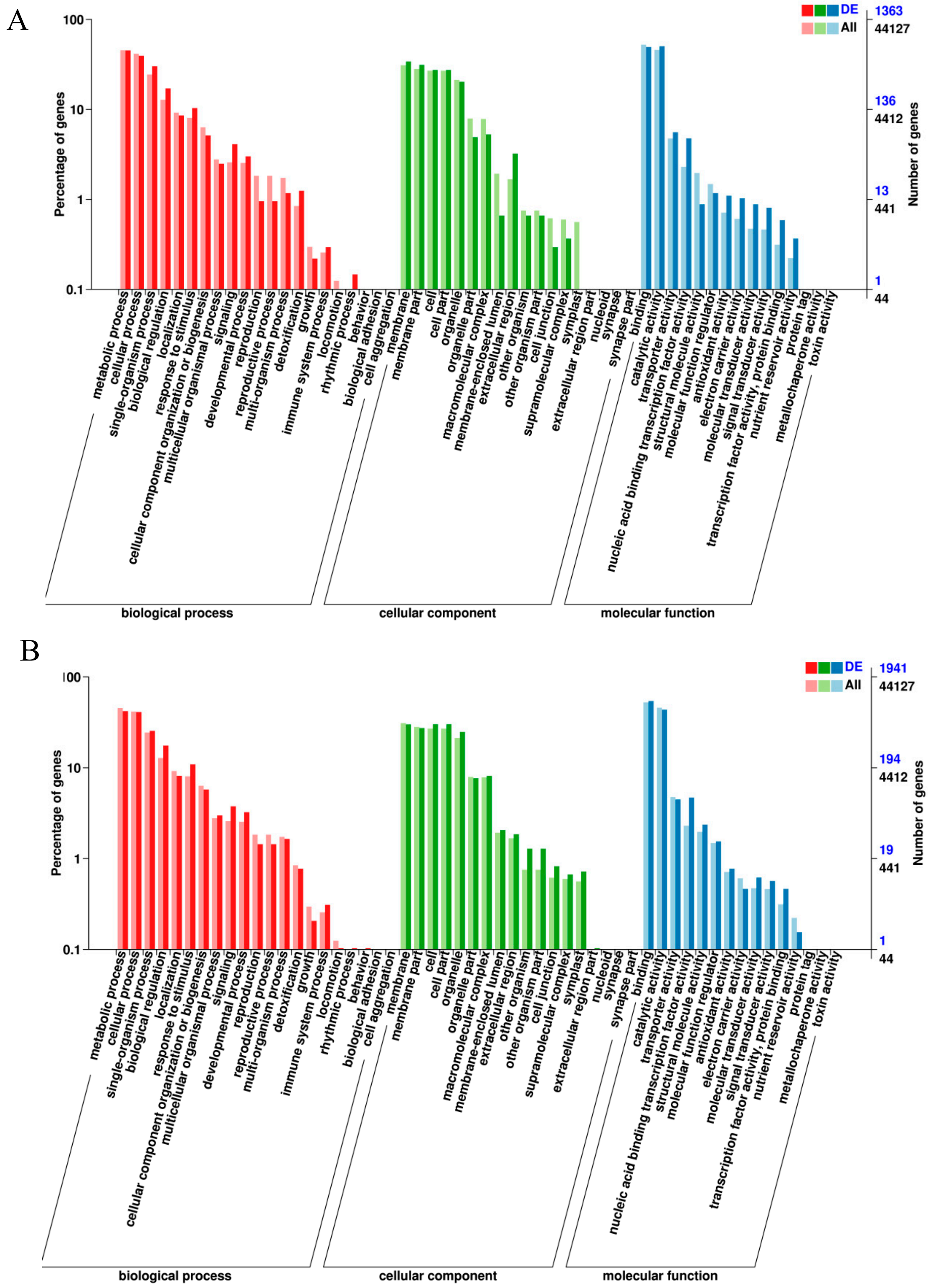

Figure S5 GO enrichment analysis of differentially expressed genes in Wulv1 (A) and Gancha4 (B) of different plant ages

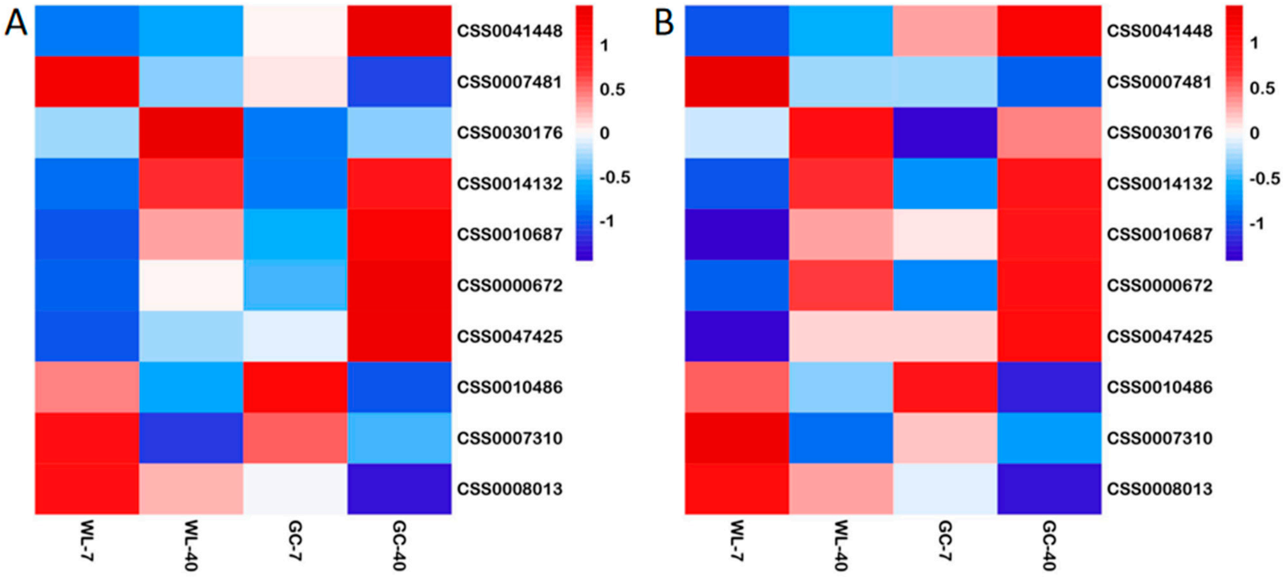

Figure S6 qPCR validation of transcriptome data. (A) Transcriptome gene expression. (B) qPCR gene expression.
